# Bibliographical Notices

**Published:** 1854-02

**Authors:** 


### BIBLIOGRAPHICAL NOTICES.

*A Treatise on the Diseases of the Eye.* By W. LAWRENCE, F. R.S., Surgeon Extraordinary to the Queen, &c., &c. *A New Edition. Edited with numerous additions, and two hundred and forty three illustrations, by* ISAAC HAYS, M. D., Surgeon to Wills Hospital; &c., &c. Philadelphia. Lea & Blanchard. 1854.

Notwithstanding the interest and importance attached to this class of diseases, and the frequency with which these affections occur, but few American treatises on Ophthalmic Medicine and Surgery have ever been offered to the public.

The Manual of Diseases of the Eye, by Dr. Littell, Surgeon to Wills Hospital, very deservedly has an extensive circulation in this country, and has been reprinted in England; with this exception, and a small work of Dr. Frick, we are entirely dependent upon British authorities for study and reference, and are therefore much gratified in being able to announce the reprint of such works as those of Walton and of Lawrence; especially when brought out under such favorable auspices.

Lawrence's book has reached a third edition in this country, and only a second in England, a fact, which, of itself, indicates the estimation of the author's labors, on this side of the Atlantic.

The American Editor has availed himself of all the recent discoveries and improvements in this department of surgery, and has added more than one hundred pages of new matter to the work.

The introductory chapter, which treats of the anatomy of the eye, has been considerably enlarged by additions from various modern authorities, and more particularly from a little work of Bowman's, a volume of great value to all who are interested in this department; from it has been taken many beautiful illustrations, elucidating minute and microscopic structures which have not appeared in any other publication.

A correct knowledge of the structure of the eye is of course the only proper basis for the study of its diseases; modern pathology, however, demands not only a mere general anatomical knowledge, but also a familiarity with the microscopic appearances of tissues, both in their normal and abnormal conditions; and this can only be obtained by practical investigations, or from the researches of able histologists.

In the subsequent chapters, Dr. Hays has given the reader the benefit of his experience and of his own views upon many topics, and has also shown great industry and research in collating, from various sources, all that is new and valuable on the subject. We may take for example the chapter on cataract, to which he has given increased value by his additions upon "Diagnosis by Catoptric Examinations," "Pathology of Cataract," "Propriety of Operating when one Eye only is Affected," "Importance of Preparatory and After Treatment," "Objection to the Operation of Depression," "Additional Remarks on the Operation by Absorption," "The Mode of Operating with Jacob's Needle," "Operation by Suction," "Diagnosis of Congenital Cataract," "Methods of Operating for," &c., &c.

In speaking of the pathology of congenital cataract, he gives the following:

"The crystalline lens is entirely devoid of vessels, and nutrition is accomplished in it, as in some of the lower orders of animals, by imbibition, or endosmose. During the early period of fœtal existence, the lens is opaque, and its capsule at the same period is vascular. From some cause, probably inflammation, which results in a deposit of lymph in the capsule, the structure of this membrane is altered, and its function, that of endosmose, impaired or arrested. Hence results an arrest of development of the lens, as this part is dependent for its nutrition on the integrity of its capsules. That this is the true view of the subject, is shown by the condition of the parts in congenital cataract. If examined soon after birth, the capsule will be found thickened by effused lymph, and the lens small. Such has been the case in every instance in which we have operated within a short period after birth. At a later period of life, a somewhat different condition is observed. The nutrition of the lens being arrested by the alteration of structure of its capsule, that body undergoes a deterioration, becomes softened and disintegrated; and, if the operation be performed towards the period of adolescence, either only a milky fluid containing some debris of the lens, with a thickened capsule, is found, or else this last is empty, and its two surfaces are in contact, forming a dense opaque membrane which it is difficult to cut with a needle, and is often insusceptible of being absorbed. Sometimes the intra-uterine inflammation of the capsule is very limited, and a single spot of lymph only is deposited on it, which does not effect the function of the membrane, or materially impair vision. In other instances, numerous opaque patches are formed, which in some rare cases, Mr. Dalrymple states, present a hard, bony or earthy surface."

After a full account of the various modes of operating for cataract by extraction, depression, and solution, the editor expresses himself as decidedly in favor of the last operation, and says:

"This operation is the one usually performed at Wills Hospital by the editor and his colleagues, and the results have been remarkably satisfactory. It is applicable, we are convinced from ample experience, to a larger number of cases than has usually been supposed, and may be successfully resorted to even in hard cataracts, by adopting the mode of operating to this condition of the lens.

This operation has the great advantage over extraction and depression, that it is far less liable to be followed by destructive inflammation; indeed in healthy individuals, when the operation is well performed, the patient suitably prepared, and proper precautions subsequently taken, the risk from inflammation is exceedingly small. It has also been urged in favor of this operation, that little manual dexterity is required for its performance. This may be true, in comparison to that required for extraction; but we protest against the inference. The merest bungler may perform it, nay, I have seen them do so and sometimes with success. But what would be the result of a large number of their operations? assuredly disastrous in a great proportion. To perform this operation well, requires skill, experience, coolness, and judgment. No one should attempt it, until he has studied well the relations of the parts involved, and has fully considered all the difficulties he may encounter, and is prepared to obviate them."

Much of the success of this operation, will depend on the surgeon keeping in mind that the capsule must be *cut;* and that in order to cut it the needle should have a knife edge. Dr. Hays recommends a needle, which we have no doubt is a valuable one, for in the first place it is short, and in the next, it has a cutting edge.

" For the proper performance of this operation, a needle, with a very keen cutting edge, is requisite. We have found it so difficult to have such an edge made on the ordinary shaped needle, that we have had an instrument especially constructed for the purpose, which contains both the advantages of a knife and a needle. This needle much resembles the ordinary iris-knife. The back is beveled, the bevel extending to one fourth of the width of the blade; the remaining three fourths constitute a knife edge. The point is made very acute, and the cutting edge extends a little over four lines from the point; below this, it gradually terminates in a round needle shank. The point is prolonged from the thickest portion of the blade, and is made to cut on both edges; below this the back is merely thined or rolled off. The whole length of the needle is about seven lines."

We might suggest an improvement upon this needle, based upon a well-known principle, viz., that straight edged instruments are much more easily sharpened than any other. Instead of having it middle-pointed, let its point be in the line of its edge, and in other respects made as directed above.

The length of this needle we would especially recommend, and believe that the preference at Wills Hospital has always been in favor of a short needle, and to this fact may, in some measure, be attributed the successful results of the operation in that institution.

There is scarcely any chapter in which we could not point out the additions and suggestions of the Editor which materially enhance the value of the book; especially should we like to bring before the reader the Editor's operations for strabismus and artificial pupil, which have justly entitled him to a high position as an ingenious operator.

But instead of dwelling longer on the general merits of the book, and its striking points of interest, we would advise a purchase of the same, to those at least, who wish a standard work on Diseases of the Eye.

The illustrations and printing are very creditable to the Publishers.

---

*Chemistry and Metallurgy, as Applied to the Study and Practice of Dental Surgery.* By A. Snowden Piggott, M. D., late Professor of Anatomy and Physiology in the Washington University of Baltimore. Lindsay & Blakiston. Philadelphia.

To treat a subject properly, with a due sense of its importance, not to fall into the error of too great conciseness on the one hand, nor into that of excessive redundance on the other, requires much judgment and discrimination. This latter mistake, the learned and full writer is, of all others, the most apt to commit, and particularly when the topic which he discusses is, from its nature, limited in its range. From an eager desire to elevate and extend what he esteems of so much value, all sources of observation and every collateral aid which can possibly be pressed into service, even where there is but little affinity between those and the main subject, are eagerly seized upon, and what, under a more proper mode of treatment, could have been soon dismissed, becomes so bulky as to require a volume to contain it.

Dr. Piggott's book, admirable as it is in many respects, partakes, in our opinion, very largely of this latter fault. As evidence of this, we need only remark that nearly one third of its contents is devoted to the chemistry of the human body and to digestion. Now considering that the application of his book, is to the *study* and *practice* of dental surgery, we cannot well see why there is associated with its useful and practical departments, matter of which the connexion is exceedingly slight and not very apparent. With quite as much reason, he might have commenced with a treatise on the anatomy and physiology of the human system. Such a mode of treating a subject, irresistibly reminds us of Diedrich Knickerbocker's History of New York, in which, to show the immense importance of his topic, he begins at the creation of the world.

Besides the fact that the first two divisions above mentioned are entirely uncalled for, we think, if it was thought proper to publish them at all, it would have shown more judgment in the writer to have preceded them with a few chapters on elementary organic chemistry. As they stand, they presume a much more intimate acquaintance with that science, than, we fear, is generally possessed by the class for which they are intended. In this opinion, we hope we are mistaken. To give our readers an opportunity of judging, however, and as a specimen, also, of the matter given us, we shall quote the first passage we light upon in turning over the leaves. It happens to be this: "*Amides* are also formed from the ammonia-salts, by the loss of an atom of water; $H_3N.C_4H_3O_3=H_2N.C_4H_3O_2+HO$. The substitution theory is inapplicable to these changes. The amides are solid, crystallizable, colorless, indifferent, volatile substances. Treated with nitrous acid, they are converted into acids with the development of ammonia.

When the amides of these acids are treated with anhydrous phosphoric acid, they lose water, and nitriles remain, which contain the radicle of the acid, and one equivalent of nitrogen in place of three of oxygen. Thus *butyramide* and phosphoric acid form hydrated acid and *butyronitrile*."

We forbear adding the chemical formula given; it is a long and tough one, which each of our readers can, as an exercise, work out for himself.

The book has four divisions. In the 1st, after describing the ultimate and proximate elements of the human body, we have a division of its substances into the two classes of azotized and non-azotized. The azotized is subdivided into exereted and histogenetic substances, which latter are treated of, under the heads of albuminous and gelatinous groups. Following these, we have described the nitrogenous basic bodies, which are not histogenetic; the non-nitrogenous acids, the haloids, the non-nitrogenous neutral bodies, and, finally, the animal pigments.

Under the 2nd head "Digestion," we have a chapter on its physiological relations, one on food, one on gastric and one on intestinal digestion, containing a description of the different secretions; as bile, pancreatic juice, intestinal juice and contents of intestinal canal and excrements.

In the third division, under the title of the chemistry of the mouth, we have an article on the teeth, with analyses of its different structures: a very full and satisfactory chapter on the saliva; one on the morbid changes of the same fluid; one on mucus and one on salivary calculi. The 4th division is devoted to the chemistry and metallurgy of the metals and earths used by dentists. In this the largest portion of the book, are described the blow-pipe and other modes of applying heat, as furnaces &c.: minute details upon the working and refining of metals used by dentists; valuable tables of gold and silver coins of different nations, all concluded by a chemical history of the materials used in, and the different methods of making incorruptible teeth.

Putting aside our objections, stated above, to the 1st and 2d. divisions, we think it but fair to state that they are extremely well written and in strict accordance with the views entertained by the latest and most reliable writers upon the subject. We cannot too highly commend the remaining portions of the work. They contain, collected from various sources and from the author's own observations, a large amount of curious and practical information, and from their intrinsic value, deserve and should command a prominent position in the library of every intelligent dentist in the country.

The work is handsomely printed and illustrated by the Publishers.

